# Association Between Severities of Obstructive Sleep Apnea and COVID-19 Outcomes

**DOI:** 10.7759/cureus.77626

**Published:** 2025-01-18

**Authors:** Lamis Alqahtani, Suzana Kano, Hanaa Bokhary, Sulafah Bahamdan, Rafah Ghazi, Shahad Abdu, Sarah Almutiri, Faris Alhejaili

**Affiliations:** 1 Faculty of Medicine, King Abdulaziz University Hospital, Jeddah, SAU; 2 Faculty of Medicine, King Abdulaziz University, Jeddah, SAU

**Keywords:** apnea, continuous positive airway pressure, covid-19, obstructive sleep apnea, sars-cov-2 virus

## Abstract

Introduction

Obstructive sleep apnea (OSA) is characterized by repetitive upper airway collapse resulting in episodes of apnea and hypopnea. Studies have shown worsened coronavirus disease 2019 (COVID-19) severity due to coexisting respiratory conditions and suggest increased severity of COVID-19 in patients with or at high risk of OSA. However, the extent of this correlation is unclear. This retrospective study aimed to evaluate the association between OSA severity and COVID-19 severity and assess the impact of continuous positive airway pressure (CPAP) compliance.

Methods

This single-center retrospective study was conducted at King Abdulaziz University Hospital (KAUH), a tertiary care center in Jeddah, Saudi Arabia. Data were collected from 62 adult patients with OSA who were diagnosed via polysomnography (PSG) and had a positive documented severe acute respiratory syndrome coronavirus 2 (SARS-CoV-2) polymerase chain reaction (PCR) test result. COVID-19 severity was categorized into mild, moderate, and severe.

Results

There was no significant correlation between OSA severity as measured by the apnea-hypopnea index (AHI), low oxyhemoglobin desaturation (LSAT), arousal index (AI), respiratory disturbance index (RDI), or the type of treatment used, including adherence to CPAP, and the outcomes of COVID-19. However, higher arousal with respiratory index (ARI) and a lower percentage of time with SpO2 < 90% (T90) values were linked to moderate COVID-19 severity with significant p-values of 0.046 and 0.007, respectively.

Conclusion

There was no significant correlation between the severity or types of OSA treatment and the severity of COVID-19. Further research including multicenter studies with bigger populations and extensive sleep study data is warranted. Understanding the OSA-COVID-19 link may improve risk stratification and patient management.

## Introduction

Obstructive sleep apnea (OSA) is defined as intermittent episodes of partial or complete upper airway obstruction during sleep despite continuous efforts to breathe normally [1-3]. Consequently, there are times when breathing is absent or diminished during sleep, referred to as apnea or hypopnea, respectively [4]. OSA is the most common sleep breathing disorder. Although it is underdiagnosed, almost one billion individuals suffer from it globally, with China exhibiting the highest prevalence rate, followed by the United States of America [4-6]. OSA prevalence in Saudi Arabia was estimated to be 4% and 1.8% among men and women, respectively [7]. The average number of apneas and hypopneas per hour of sleep is known as the apnea-hypopnea index (AHI), which is frequently used to evaluate the severity of OSA and treatment efficacy [8]. Continuous positive airway pressure (CPAP) is the cornerstone of moderate to severe OSA treatment. However, the benefits of ventilation mainly depend on patient compliance and commitment to the treatment plan [9].

OSA has also been hypothesized to increase coronavirus disease 2019 (COVID-19) severity [6]. COVID-19 is a severe respiratory illness induced by severe acute respiratory syndrome coronavirus 2 (SARS-CoV-2) infection [10,11] and was declared a pandemic on March 11, 2020, by the World Health Organization (WHO) [12,13]. Clinical manifestations of COVID-19 range from asymptomatic or mild to debilitating disease that can result in death [14]. Early findings suggested that coexisting respiratory illnesses increase the likelihood of COVID-19 severity [14-16]. The severity of COVID-19 is reflected by the percentage of patients requiring hospitalization and intensive care unit (ICU) admission [17]. Patients with severe COVID-19 and those with preexisting OSA share common risk factors, including male sex, high body mass index (BMI), and older age [18,19].

Multiple attempts have been made to analyze the correlation between OSA and COVID-19, including an observational retrospective study suggesting that the chances of severe COVID-19 occurrence should be considered in patients at high risk of OSA [20]. Another study in Finland found that although OSA does not affect the risk of contracting COVID-19, it is associated with a higher risk of hospitalization [21]. Similarly, Kravitz et al. found a dose-response relationship between OSA severity and increased risk of hospitalization due to COVID-19 [22]. Moreover, the presence of OSA doubles the risk of respiratory failure according to both Cade et al. [23] and Maas et al. [24]. Hariyanto et al. also reported a significant association with the use of mechanical ventilation and ICU admissions [25]. In terms of mortality, OSA is reportedly an independent factor associated with death due to COVID-19 [26]. In addition, Cade et al. found that having OSA increased the mortality odds by 1.79 [23]. However, studies by Cade et al. [23] and Mashaqi et al. [27] showed no statistical significance for ICU admission or the length of stay (LOS) for patients with OSA.

Further research conducted at Isala Hospital in the Netherlands revealed that only low oxyhemoglobin desaturation (LSAT) parameters were significantly correlated with the severity of COVID-19. However, the degree of OSA based on AHI, oxygen desaturation index, respiratory disturbance index (RDI), and OSA treatment were not found to be risk factors [2]. Yet again, a retrospective study found that CPAP was not consistently linked to worse outcomes in patients hospitalized because of COVID-19 [28].

Although several studies have been conducted, the extent to which the degree of OSA correlates with COVID-19 severity remains unclear, with no studies having been conducted on this topic in Saudi Arabia to date. Further, little research has been conducted on the impact of CPAP compliance on COVID-19 outcomes. Therefore, this retrospective study conducted at King Abdulaziz University Hospital (KAUH), Saudi Arabia, aimed to evaluate whether the degree of OSA was associated with COVID-19 severity. The secondary aim was to explore the impact of CPAP compliance on the outcomes of COVID-19.

## Materials and methods

Study population and setting

This study was a single-center retrospective record review conducted at KAUH, a tertiary care center in Jeddah, Saudi Arabia. Electronic medical records between December 2013 and December 2020 at the division of Sleep Medicine and Research Centre (SMRC) were accessed. This study was approved by the Institutional Review Board of KAUH (reference number: 82-22).

We collected and analyzed the data of 984 patients who underwent polysomnography (PSG), the gold diagnostic modality for OSA [29], to obtain contact numbers of adult (≥18 years) patients who had been diagnosed with OSA (AHI > 5 events/hour) between December 2013 and December 2020. Of the 984 patients, 787 were excluded for missing data, wrong phone numbers, or unwillingness to participate. Subsequently, patients with no history of positive SARS-CoV-2 results on quantitative polymerase chain reaction (PCR) test, missing PSG data, or duplications were excluded. The final sample size was 62.

Inclusion Criteria

Participants aged 18 years or older, those with a confirmed diagnosis of OSA based on PSG results, those with a history of COVID-19 infection confirmed through a positive PCR test, and those who provided informed consent via electronic Google Forms (Google, Inc., Mountain View, CA) were included.

Exclusion Criteria

Participants under the age of 18 years, those with missing or incomplete baseline or follow-up data, and those who declined to participate in the study or withdrew consent at any stage were excluded.

COVID-19 status

To take advantage of the large number of patients referred to the SMRC from other regions, including Jeddah City, we used online patient-filled Google Forms distributed via WhatsApp, a multiplatform messaging application. This approach ensured a broad reach, facilitated the confirmation of COVID-19 infection history, and obtained informed consent. Participants were informed of the study goals and assured of the privacy of their responses before providing data.

The form consisted of two sections. The first section collected information on smoking history, OSA treatment (CPAP or surgical), CPAP compliance (>4 hours a night for 70% of nights), and the number of COVID-19 vaccination doses received before infection. The second section focused on the first COVID-19 illness course, including questions to assess the severity of COVID-19 infection. We used the COVID-19 WHO Clinical Progression Scale. The scale categorizes severity as follows: mild (asymptomatic or mild symptoms that did not require hospitalization), moderate (hospitalized), and severe (hospitalized in the ICU). Additional details such as length of stay (LOS) and oxygenation requirements were also collected.

While this method enabled the collection of detailed COVID-19 outcome data in a cost-effective and time-efficient manner, we acknowledge the potential for recall bias, particularly for patients with mild symptoms or those infected during earlier stages of the pandemic when milder variants were more prevalent. To minimize this, we focused on documented positive PCR test results rather than relying solely on self-reported symptoms. This approach aimed to reduce the impact of recall bias and improve the accuracy of the reported infection status. This limitation is addressed in the discussion section.

OSA status

Sex, age, height, weight, comorbidities, and neck circumference were recorded as the baseline data. Moreover, the extracted PSG results consisted of AHI, RDI, arousal index (AI), arousal with respiratory index (ARI), percentage of time with SpO2 < 90% (T90), and LSAT.

AHI is defined as the total number of apnea and hypopnea events per hour recorded during sleep, with apnea defined as complete cessation of breathing for ≥10 seconds and hypopnea as a decrease of ≥30% in airflow accompanied by a decrease in oxygen saturation of ≥4%. Depending on the AHI score, participants' sleep apnea was divided into three groups: mild OSA (5-14.9 events/hour), moderate OSA (15-29.9 events/hour), and severe OSA (>30 events/hour).

Another OSA measurement was RDI, which is similar to AHI but considers respiratory effort-related arousals, which is an event causing arousal or a decrease in oxygen saturation without qualifying as apnea or hypopnea. The total number of arousals per hour of total sleep time (TST) is AI. ARI is the number of arousals per hour of the TST correlated with respiratory events.

Statistical analysis

Microsoft Excel (Microsoft Corp., Redmond, WA) was used for data entry, and data analysis was performed using Jamovi. Qualitative data are presented as frequencies and percentages, while quantitative variables are represented as medians and interquartile ranges (IQRs). A multinomial logistic regression analysis was used to determine the association between the degree of OSA and COVID-19 severity. The effects of OSA treatment type, smoking, and vaccination status on COVID-19 severity (mild, moderate, or severe) were assessed using the chi-squared test. Pearson's and Spearman's correlation analyses were used to evaluate the correlation between the degree of severity and LOS. The statistical significance level was set at p < 0.05.

## Results

Between 2013 and 2020, 984 patients were diagnosed with OSA after undergoing PSG at the SMRC. However, 194 were enrolled after accounting for data duplication and the availability of COVID-19 information. Of these, only 62 fulfilled the inclusion and exclusion criteria for further analysis.

The majority of patients were men, 38 (61.3%) had never smoked, and approximately 43 (70%) had been vaccinated at least once before contracting COVID-19. Obesity was the most common comorbidity, followed by diabetes mellitus and hypertension (Table 1). Regarding sleep study parameters, the median AHI was 18.6 events/hour, with severity levels almost evenly distributed between mild, moderate, and severe patients. Overall, 40 (75%) patients reported experiencing daytime sleepiness and 33 (>60%) complained of snoring (Table 2), while 31 (50%) recorded an LSAT of ≥84 and a T90 of ≤1.2% (Table 3).

**Table 1:** Patients' characteristics Data are presented as numbers and percentages or medians and IQRs. #: available for 40 patients only, IQR: interquartile range, BMI: body mass index, COVID-19: coronavirus disease 2019

Characteristics	Number (%)
Age, years (median (IQR))	48 (40, 52.8)
Sex	
Female	17 (27.4 %)
Male	45 (72.6%)
Nationality	
Saudi	55 (88.7%)
Non-Saudi	7 (11.3%)
BMI, kg/m^2^ (median (IQR))	33.1 (29.4, 43.1)
Normal weight	1 (1.6%)
Overweight	16 (25.8%)
Obesity class 1	19 (30.6%)
Obesity class 2	8 (12.9%)
Obesity class 3	18 (29.0%)
Neck circumference (median (IQR))^#^	15.5 (15, 16.5)
Smoking status	
Current smokers	15 (24.2%)
Former smokers	9 (14.5%)
Never smoked	38 (61.3%)
COVID-19 vaccination status pre-infection	
Zero dose	19 (30.6%)
One dose	8 (12.9%)
Two or more doses	35 (56.5%)
Comorbidities	
Obesity	45 (72.6%)
Diabetes mellitus	25 (40.3%)
Hypertension	22 (35.5%)
Asthma	12 (19.4%)
Allergic rhinitis	12 (19.4%)
Gastroesophageal reflux disease	8 (14.5%)
Dyslipidemia	7 (11.3%)
Hypothyroidism	6 (9.7%)
Deviated nasal septum	3 (4.8%)
Anxiety	2 (3.2%)
Chronic obstructive pulmonary disease	1 (1.6%)

**Table 2:** OSA characteristics Data are presented as numbers and percentages or median and IQRs. AHI: apnea-hypopnea index, IQR: interquartile range, OSA: obstructive sleep apnea

	Median (IQR)	Range
AHI	18.6 (10.4, 33.3)	5.3-86.2
OSA severity (AHI)	Number	%
Mild OSA	24/62	38.7%
Moderate OSA	19/62	30.6%
Severe OSA	19/62	30.6%
OSA symptoms		
Daytime sleepiness	40/53	75.5%
Snoring	33/52	63.5%
Apnea episodes	14/51	27.5%
Insomnia	14/62	22.6%
Abrupt awakenings accompanied by gasping	8/51	15.7%
Headache	8/51	15.7%

**Table 3:** Sleep study parameters Data are presented as medians, IQR, and range. IQR: interquartile range, LSAT: low oxyhemoglobin desaturation, RDI: respiratory disturbance index, AI: arousal index, ARI: arousal with respiratory index, T90: percentage of time with SpO2 < 90%

	Median (IQR)	Range
LSAT (n = 62)	84.0 (72.75-88.25)	30-94
RDI (n = 61)	18.4 (10.2-33.15)	5-80
AI (n = 59)	19.2 (10.70-41.0)	0-110
ARI (n = 58)	6.2 (2.23-19.83)	0-76.4
T90 (n = 56)	1.2 (0.20-13.38)	0-95

COVID-19 severity in patients was classified as mild, moderate, or severe based on the COVID-19 WHO Clinical Progression Scale. In the univariate multinomial regression analysis, there was no statistically significant association between AHI, LSAT, RDI, or AI and COVID-19 severity in patients with moderate or severe infection. However, ARI and T90 were associated with moderate COVID-19 severity, with an odds ratio (95% confidence interval) of 1.0382 (1.001-1.077) (p = 0.046) and 1.0390 (1.01-1.068) (p = 0.007), respectively (Table 4).

**Table 4:** Multinomial logistic regression analysis for COVID-19 severity categorized as mild, moderate, and severe p-values compare the independent variables of hospitalized COVID-19 patients with reference patients. Reference patients were COVID-19 outpatients with mild disease. p < 0.05 was considered statistically significant. AHI: apnea-hypopnea index, LSAT: low oxyhemoglobin desaturation, RDI: respiratory disturbance index, AI: arousal index, ARI: arousal with respiratory index, T90: percentage of time with SpO2 < 90%, OR: odds ratio, confidence interval, SE: standard error

Independent variables	Coefficient	SE	Moderate disease	p-value	Coefficient	SE	Severe disease	p-value
			OR (95% CI)				OR (95% CI)	
AHI	0.0203	0.015	1.021 (0.991-1.051)	0.181	0.0347	0.026	1.035 (0.983-1.09)	0.188
LSAT	-0.0358	0.0243	0.965 (0.92-1.01)	0.141	-0.0327	0.0421	0.968 (0.891-1.05)	0.437
RDI	0.0239	0.0158	1.0242 (0.993-1.056)	0.130	0.0393	0.0275	1.04 (0.986-1.098)	0.153
AI	0.01052	0.0141	1.0106 (0.983-1.039)	0.456	0.00857	0.0253	1.01 (0.96-1.06)	0.735
ARI	0.0375	0.0188	1.0382 (1.001-1.077)	0.046	0.0298	0.0304	1.0303 (0.971-1.094)	0.327
T90	0.0382	0.0142	1.0390 (1.01-1.068)	0.007	0.0207	0.0245	1.0209 (0.973-1.071)	0.399

CPAP was the primary treatment choice for OSA, with roughly similar numbers of adherent and non-adherent patients. Neither the treatment type nor adherence to CPAP was significant. The smoking status differed considerably among the three groups. Patients who never smoked were more prevalent in the mild group (34 (64.2%)), whereas all patients who experienced severe COVID-19 symptoms were smokers (Table 5).

**Table 5:** Comparison between the degree of COVID-19 severity groups Data are presented as numbers and percentages, and the chi-squared test was used to compare the three groups. OSA: obstructive sleep apnea, CPAP: continuous positive airway pressure, BIPAP: bilevel positive airway pressure, NA: not applicable, *: includes outpatients and missing

	COVID-19 severity			Total (N = 62)	ꭓ2	p-value
	Mild (n = 53)	Moderate (n = 7)	Severe (n = 2)			
OSA treatment type						
Missing	2	1	0	3		
CPAP	21 (41.2%)	4 (66.7%)	1 (50.0%)	26 (44.1%)	4.381	0.9201
Surgical	9 (17.6%)	1 (16.7%)	0 (0.0%)	10 (16.9%)		
BIPAP	1 (2.0%)	0 (0.0%)	0 (0.0%)	1 (1.7%)		
CPAP and surgery	5 (9.8%)	1 (16.7%)	0 (0.0%)	6 (10.2%)		
None	11 (21.6%)	0 (0.0%)	1 (50.0%)	12 (20.3%)		
Others	4 (7.8%)	0 (0.0%)	0 (0.0%)	4 (6.8%)		
Surgery type						
Missing	3	0	0	3		
No surgery	37 (74.0%)	5 (71.4%)	1 (50.0%)	43 (72.9%)	5.280	0.508
Bariatric	5 (10.0%)	2 (28.6%)	0 (0.0%)	7 (11.9%)		
ENT	7 (14.0%)	0 (0.0%)	1 (50.0%)	8 (13.6%)		
Maxillofacial	1 (2.0%)	0 (0.0%)	0 (0.0%)	1 (1.7%)		
CPAP compliance						
Missing	6	1	0	7		
NA	21	1	1	23		
Compliant	11 (42.3%)	3 (60.0%)	1 (100.0%)	15 (46.9%)	2.542	0.428
Non-compliant	15 (57.7%)	2 (40.0%)	0 (0.0%)	17 (53.1%)		
Oxygen therapy						
NA*	44	1	0	45		
Noninvasive ventilation	2 (22.2%)	4 (66.7%)	1 (50.0%)	7 (41.2%)	39.092	0.019
Mechanical ventilation	0 (0.0%)	0 (0.0%)	1 (50.0%)	2 (5.9%)		
Not needed	7 (77.8%)	2 (33.3%)	0 (0.0%)	9 (52.9%)		
Smoking						
Cigarettes	4 (7.5%)	0 (0.0%)	2 (100.0%)	6 (9.7%)	22.939	0.011
Sheesha	4 (7.5%)	2 (28.6%)	0 (0.0%)	6 (9.7%)		
Cigarettes and sheesha	1 (1.9%)	0 (0.0%)	0 (0.0%)	1 (1.6%)		
E-cigarettes	2 (3.8%)	0 (0.0%)	0 (0.0%)	2 (3.2%)		
Stopped smoking	8 (15.1%)	1 (14.3%)	0 (0.0%)	9 (14.5%)		
Never smoke	34 (64.2%)	4 (57.1%)	0 (0.0%)	38 (61.3%)		
Vaccination status						
Not vaccinated	14 (26.4%)	4 (57.1%)	1 (50.0%)	19 (30.6%)	5.637	0.465
Vaccinated	39 (73.6%)	3 (42.9%)	1 (50.0%)	43 (69.4%)		

## Discussion

This study examined the relationship between OSA severity and compliance with CPAP therapy in COVID-19 progression. Surprisingly, our analyses revealed no statistically significant associations between most OSA parameters or OSA treatment types and COVID-19 severity.

Regarding sleep study parameters, our results showed no statistically significant associations between the AHI, RDI, and COVID-19 severity in patients with moderate or severe infections, which confirms the results from a study by Ho et al. [2]. However, they found LSAT to be a significant risk factor for COVID-19 severity when COVID-19 outcomes were adjusted and merged into two categories (non-hospitalized or hospitalized and ICU or death), criteria that do not adhere to those described by WHO criteria used in this study. Interestingly, we found a significant association between high ARI and low T90 and worse COVID-19 outcomes in patients with OSA. These two parameters have not been used in previous studies on COVID-19. However, Budhiraja et al. found that T90 was significantly associated with moderate to severe OSA [30]. This suggests that T90 and ARI indirectly reflect the severity of OSA in patients with COVID-19.

Additionally, the present study examined whether the type of OSA treatment (medical versus surgical) and CPAP compliance were associated with COVID-19 severity and found no statistically significant relationship between them. Our results align with a recent retrospective study conducted in Spain that showed a non-significant link between OSA treated with CPAP and worse outcomes in patients hospitalized owing to COVID-19 (p = 0.303) [28]. In contrast, Genzor et al. revealed that patients with OSA who adhered to CPAP therapy were less likely to experience a severe course of COVID-19 or death than patients with OSA who were non-adherent to treatment [31]. This could be explained by the fact that adherence to CPAP improves long-term morbidity, such as cardiovascular disease, thus improving COVID-19 outcomes. The differences in results may be related to the timing of CPAP initiation, which should be considered.

This study had some limitations. As with any retrospective record review, not all required data were documented. This was amplified by the fact that the SMRC serves not only patients at KAUH but also the entire western region of Saudi Arabia. As such, follow-up of these cases was challenging. At the same time, we believe it provides a reasonably broad representation of the Saudi population. Despite this, we recognize that our findings might not be fully applicable to populations in other regions with different healthcare systems and demographic characteristics.

Additionally, the retrospective nature of the study and reliance on patient-reported data for COVID-19 severity may have introduced recall bias, particularly for those with mild symptoms or infections during earlier stages of the pandemic, when milder variants were more prevalent. To minimize this, we focused on documented positive PCR test results rather than relying solely on self-reported symptoms. This approach strengthens the reliability of the data despite potential recall bias. Furthermore, by applying strict inclusion and exclusion criteria, we ensured that the study population represented a specific group of patients, enhancing internal validity. However, this also resulted in a limited sample size, which may reduce the statistical power to detect significant associations, such as AHI, which was the main parameter in other studies. Despite this limitation, our study provides valuable insights into the potential link between OSA and COVID-19 outcomes, setting a foundation for future research, which should focus on conducting a multicenter study with a larger population and collecting comprehensive sleep study data to facilitate better risk stratification and patient management strategies.

## Conclusions

We did not find a statistically significant association between the severities of OSA and COVID-19 outcomes when OSA was represented by AHI, LSAT, AI, and RDI. Neither CPAP nor any other treatment had a significant effect on the severity of OSA. Meanwhile, high ARI and low T90 values were associated with moderate COVID-19 severity.

Our findings suggest that in individuals with suspected or confirmed COVID-19, OSA should be considered a risk factor for acquiring a severe form of the disease.

## References

[1] Kar A, Saxena K, Goyal A (2021). Assessment of obstructive sleep apnea in association with severity of COVID-19: a prospective observational study. Sleep Vigil.

[2] Ho JP, Donders HC, Zhou N, Schipper K, Su N, de Lange J (2021). Association between the degree of obstructive sleep apnea and the severity of COVID-19: an explorative retrospective cross-sectional study. PLoS One.

[3] Grasselli G, Tonetti T, Protti A (2020). Pathophysiology of COVID-19-associated acute respiratory distress syndrome: a multicentre prospective observational study. Lancet Respir Med.

[4] Malhotra A, White DP (2002). Obstructive sleep apnoea. Lancet.

[5] Benjafield AV, Ayas NT, Eastwood PR (2019). Estimation of the global prevalence and burden of obstructive sleep apnoea: a literature-based analysis. Lancet Respir Med.

[6] Chung F, Waseem R, Pham C (2021). The association between high risk of sleep apnea, comorbidities, and risk of COVID-19: a population-based international harmonized study. Sleep Breath.

[7] Wali SO, Abalkhail B, Krayem A (2017). Prevalence and risk factors of obstructive sleep apnea syndrome in a Saudi Arabian population. Ann Thorac Med.

[8] Asghari A, Mohammadi F, Kamrava SK, Tavakoli S, Farhadi M (2012). Severity of depression and anxiety in obstructive sleep apnea syndrome. Eur Arch Otorhinolaryngol.

[9] Mutti C, Azzi N, Soglia M, Pollara I, Alessandrini F, Parrino L (2020). Obstructive sleep apnea, CPAP and COVID-19: a brief review. Acta Biomed.

[10] Garg S, Kim L, Whitaker M (2020). Hospitalization rates and characteristics of patients hospitalized with laboratory-confirmed coronavirus disease 2019 - COVID-net, 14 states, March 1-30, 2020. MMWR Morb Mortal Wkly Rep.

[11] Salje H, Tran Kiem C, Lefrancq N (2020). Estimating the burden of SARS-CoV-2 in France. Science.

[12] Zhu N, Zhang D, Wang W (2020). A novel coronavirus from patients with pneumonia in China, 2019. N Engl J Med.

[13] Wilder-Smith A, Chiew CJ, Lee VJ (2020). Can we contain the COVID-19 outbreak with the same measures as for SARS?. Lancet Infect Dis.

[14] Wu Z, McGoogan JM (2020). Characteristics of and important lessons from the coronavirus disease 2019 (COVID-19) outbreak in China: summary of a report of 72 314 cases from the Chinese Center for Disease Control and Prevention. JAMA.

[15] Ramanathan K, Shekar K, Ling RR (2021). Extracorporeal membrane oxygenation for COVID-19: a systematic review and meta-analysis. Crit Care.

[16] Guan WJ, Ni ZY, Hu Y (2020). Clinical characteristics of coronavirus disease 2019 in China. N Engl J Med.

[17] Li X, Xu S, Yu M (2020). Risk factors for severity and mortality in adult COVID-19 inpatients in Wuhan. J Allergy Clin Immunol.

[18] Zheng Z, Peng F, Xu B (2020). Risk factors of critical & mortal COVID-19 cases: a systematic literature review and meta-analysis. J Infect.

[19] Chidambaram V, Tun NL, Haque WZ (2020). Factors associated with disease severity and mortality among patients with COVID-19: a systematic review and meta-analysis. PLoS One.

[20] Tufik S, Gozal D, Ishikura IA, Pires GN, Andersen ML (2020). Does obstructive sleep apnea lead to increased risk of COVID-19 infection and severity?. J Clin Sleep Med.

[21] Strausz S, Kiiskinen T, Broberg M (2021). Sleep apnoea is a risk factor for severe COVID-19. BMJ Open Respir Res.

[22] Kravitz MB, Yakubova E, Yu N, Park SY (2021). Severity of sleep apnea and COVID-19 illness. OTO Open.

[23] Cade BE, Dashti HS, Hassan SM, Redline S, Karlson EW (2020). Sleep apnea and COVID-19 mortality and hospitalization. Am J Respir Crit Care Med.

[24] Maas MB, Kim M, Malkani RG, Abbott SM, Zee PC (2021). Obstructive sleep apnea and risk of COVID-19 infection, hospitalization and respiratory failure. Sleep Breath.

[25] Hariyanto TI, Kurniawan A (2021). Obstructive sleep apnea (OSA) and outcomes from coronavirus disease 2019 (COVID-19) pneumonia: a systematic review and meta-analysis. Sleep Med.

[26] Cariou B, Hadjadj S, Wargny M (2020). Phenotypic characteristics and prognosis of inpatients with COVID-19 and diabetes: the CORONADO study. Diabetologia.

[27] Mashaqi S, Lee-Iannotti J, Rangan P, Celaya MP, Gozal D, Quan SF, Parthasarathy S (2021). Obstructive sleep apnea and COVID-19 clinical outcomes during hospitalization: a cohort study. J Clin Sleep Med.

[28] Sampol J, Sáez M, Martí S, Pallero M, Barrecheguren M, Ferrer J, Sampol G (2022). Impact of home CPAP-treated obstructive sleep apnea on COVID-19 outcomes in hospitalized patients. J Clin Sleep Med.

[29] Kapur VK, Auckley DH, Chowdhuri S, Kuhlmann DC, Mehra R, Ramar K, Harrod CG (2017). Clinical practice guideline for diagnostic testing for adult obstructive sleep apnea: an American Academy of Sleep Medicine clinical practice guideline. J Clin Sleep Med.

[30] Budhiraja R, Javaheri S, Parthasarathy S, Berry RB, Quan SF (2019). The association between obstructive sleep apnea characterized by a minimum 3 percent oxygen desaturation or arousal hypopnea definition and hypertension. J Clin Sleep Med.

[31] Genzor S, Prasko J, Mizera J (2022). Risk of severe COVID-19 in non-adherent OSA patients. Patient Prefer Adherence.

